# Author Correction: Kalium channelrhodopsins effectively inhibit neurons

**DOI:** 10.1038/s41467-024-50250-y

**Published:** 2024-07-19

**Authors:** Stanislav Ott, Sangyu Xu, Nicole Lee, Ivan Hong, Jonathan Anns, Danesha Devini Suresh, Zhiyi Zhang, Xianyuan Zhang, Raihanah Harion, Weiying Ye, Vaishnavi Chandramouli, Suresh Jesuthasan, Yasunori Saheki, Adam Claridge-Chang

**Affiliations:** 1 Program in Neuroscience and Behavioral Disorders, Duke-NUS Medical School, Singapore, Singapore; 2 Institute for Molecular and Cell Biology, A*STAR Agency for Science, Technology and Research, Singapore, Singapore; 3 School of Biological Sciences and Institute for Life Sciences, University of Southampton, Southampton, UK; 4 Lee Kong Chian School of Medicine, Nanyang Technological University, Singapore, Singapore; 5 Department of Pharmacy, National University of Singapore, Singapore, Singapore; 6 Department of Physiology, National University of Singapore, Singapore, Singapore

**Keywords:** Motor neuron, Neuronal physiology

Correction to: *Nature Communications* 10.1038/s41467-024-47203-w, published online 24 April 2024

In this article, one affiliation for Adam Claridge-Chang was incorrectly given as Department of Pharmacy, National University of Singapore, Singapore, Singapore but should have been Department of Physiology, National University of Singapore, Singapore, Singapore. The original article has been corrected.

