# Supplementary material for: Kalium channelrhodopsins effectively inhibit neurons
Source: Nat Commun. 2024 Apr 24;15:3480. doi: 10.1038/s41467-024-47203-w (PMC11043423; doi:10.1038/s41467-024-47203-w)
Supplement: Supplementary file 1 — Supplementary Information [file 41467_2024_47203_MOESM1_ESM.pdf]

Supplementary Information

Kalium channelrhodopsins effectively inhibit neurons

Stanislav Ott [1], Sangyu Xu [2], Nicole Lee [1], Ivan Hong Hee Kean [1], Jonathan Anns [2, 3], Danesha Devini Suresh [1], Zhiyi Zhang [1], Xianyuan Zhang [1], Raihanah Harion [4], Weiying Ye [5], Vaishnavi Chandramouli [4], Suresh Jesuthasan [4], Yasunori Saheki [4], Adam Claridge-Chang [1, 2, 4, 6]

1. Program in Neuroscience and Behavioral Disorders, Duke-NUS Medical School, Singapore
2. Institute for Molecular and Cell Biology, A*STAR Agency for Science, Technology and Research, Singapore
3. School of Biological Sciences and Institute for Life Sciences, University of Southampton, UK
4. Lee Kong Chian School of Medicine, Nanyang Technological University, Singapore
5. Department of Pharmacy, National University of Singapore, Singapore
6. Correspondence. claridge-chang.adam@duke-nus.edu.sg

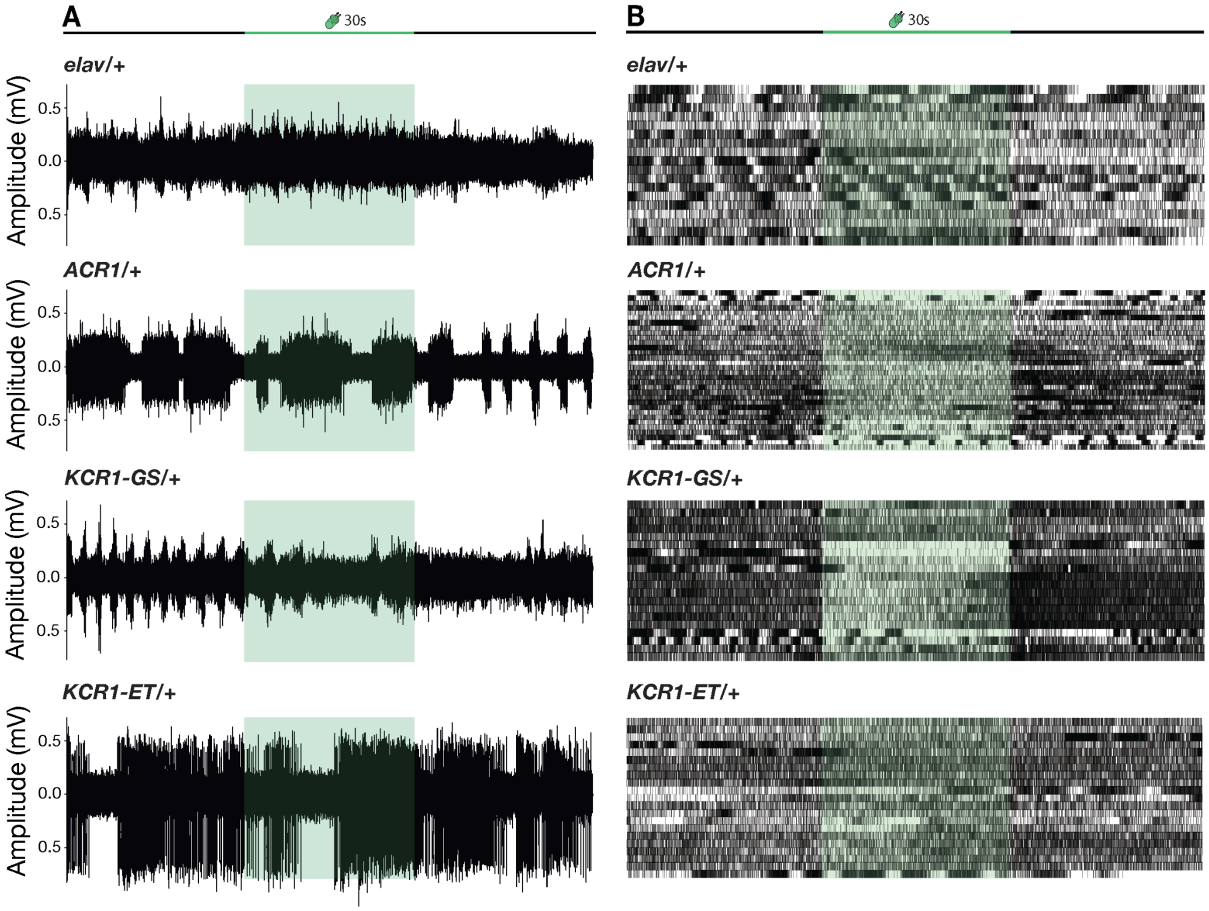

Figure S1: Effects of illumination on larval abdominal nerves in control genotypes. Representative recordings (**A**) and raster summaries (**B**) of action potential occurrences for sibling control genotypes. Action potentials were recorded from the fly larval abdominal nerve 3. For all representative traces n = 1 biologically independent sample over 1 independent experiment. For all raster summaries n = 3 biologically independent samples over 3 independent experiments. Source data are provided as a Source Data file.

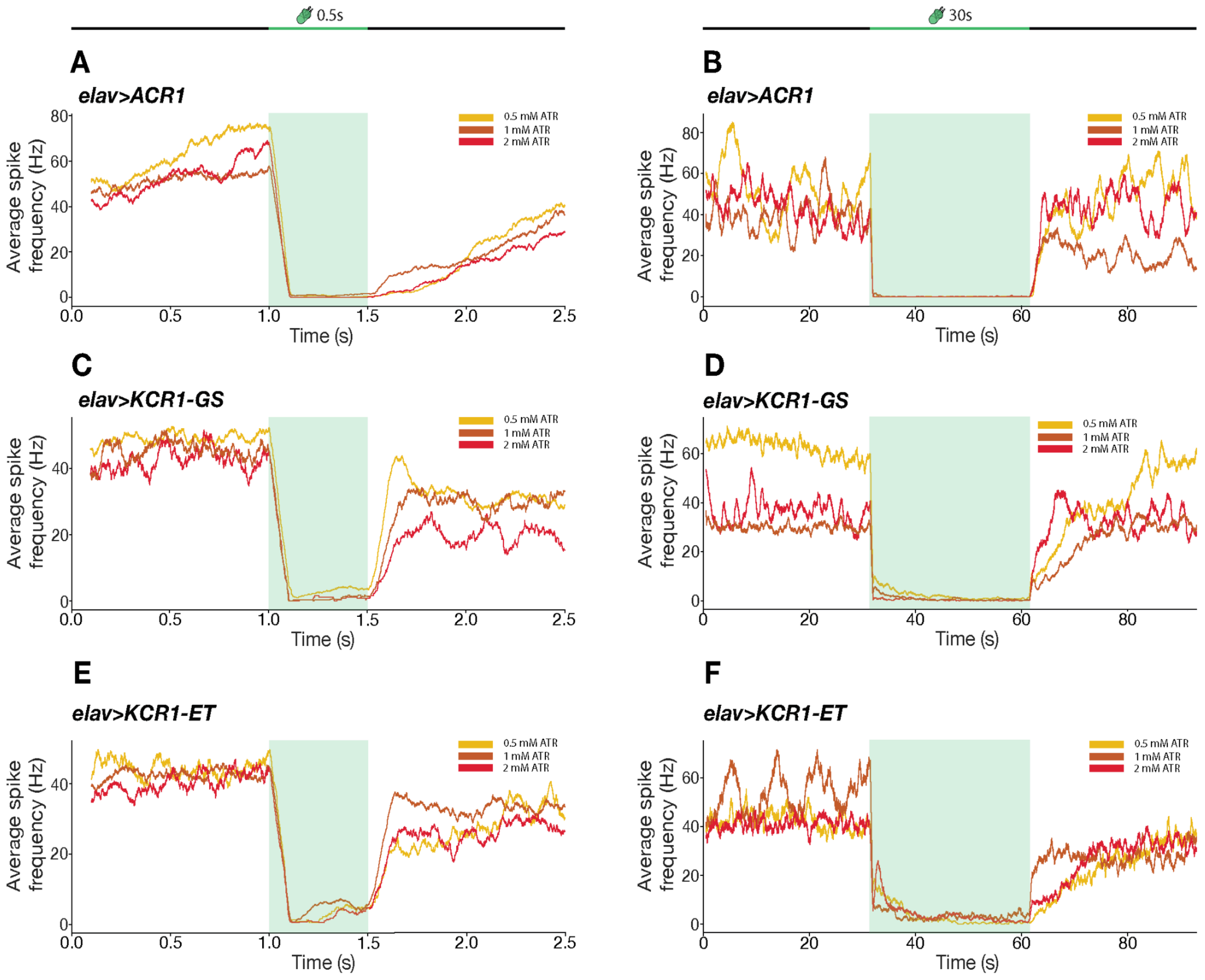

Figure S2: Inhibition of spontaneous spikes at different ATR concentrations
Average spike frequency comparisons for *Drosophila* larvae reared on food containing different ATR concentrations. Experiments with 0.5 s optogenetic actuation (40 µW/mm^2) (**left: A, C, E**) and 30s actuation (40 µW/mm^2) periods (**right: B, D, F**) are shown. ACR1-induced silencing was slightly more potent than KCR-induced silencing at all ATR concentrations. Action potential recovery after 0.5 s actuation was slower in ACR-expressing larvae as compared to KCR-expressing larvae. For all genotypes and conditions n = 3 biologically independent samples over 3 independent experiments. Additional statistical information for all panels is presented in Supplementary Dataset 1. Source data are provided as a Source Data file.

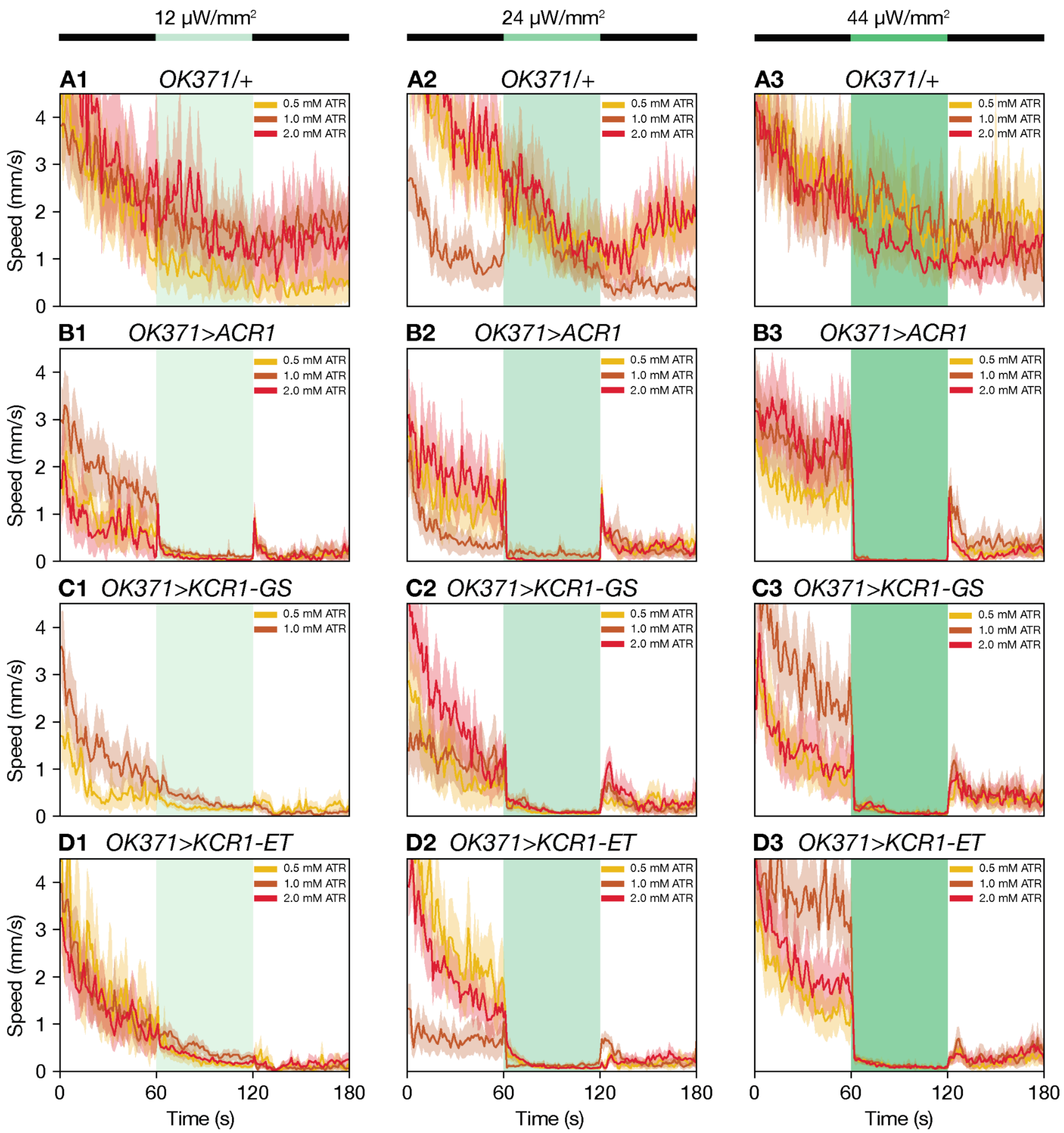

Figure S3: Walking inhibition at different ATR concentrations and light intensities
Locomotor impairment comparisons for different ATR and light dosages. Each line plot shows the average performance with a 95% CI ribbon. The schematic (top) and the green rectangle in each panel indicates the time of illumination.

A: Locomotor activity of the driver control before, during and after light actuation.

A1. *OK371/+* 0.5 mM ATR, 12 μW/mm^2 n = 39 biologically independent animals over 2 independent experiments. *OK371/+* 1 mM ATR, 12 μW/mm^2 n = 66 biologically independent animals over 3 independent experiments. *OK371/+* 2 mM ATR, 12 μW/mm^2 n = 33 biologically independent animals over 2 independent experiments.

OK371/+ 0.5 mM ATR, 24 μW/mm^2 n = 59 biologically independent animals over 3 independent experiments. *OK371/+* 1 mM ATR, 24 μW/mm^2 n = 76 biologically independent animals over 3 independent experiments.

A2. *OK371/+* 2 mM ATR, 24 μW/mm^2 n = 45 biologically independent animals over 2 independent experiments. *OK371/+* 0.5 mM ATR, 44 μW/mm^2 n = 39 biologically independent animals over 2 independent experiments.

A3. *OK371/+* 1 mM ATR, 44 μW/mm^2 n = 36 biologically independent animals over 2 independent experiments. *OK371/+* 2 mM ATR, 44 μW/mm^2 n = 68 biologically independent animals over 3 independent experiments.

B. Locomotor activity of *OK371>ACR1* flies before, during and after light actuation.

B1. *OK371>ACR1* 0.5 mM ATR, 12 μW/mm^2 n = 41 biologically independent animals over 2 independent experiments. *OK371>ACR1* 1 mM ATR, 12 μW/mm^2 n = 65 biologically independent animals over 3 independent experiments. *OK371>ACR1* 2 mM ATR, 12 μW/mm^2 n = 20 biologically independent animals over 1 independent experiment.

B2. *OK371>ACR1* 0.5 mM ATR, 24 μW/mm^2 n = 44 biologically independent animals over 2 independent experiments. *OK371>ACR1* 1 mM ATR, 24 μW/mm^2 n = 72 biologically independent animals over 2 independent experiments. *OK371>ACR1* 2 mM ATR, 24 μW/mm^2 n = 44 biologically independent animals over 2 independent experiments.

B3. *OK371>ACR1* 0.5 mM ATR, 44 μW/mm^2 n = 66 biologically independent animals over 3 independent experiments. *OK371>ACR1* 1 mM ATR, 44 μW/mm^2 n = 70 biologically independent animals over 3 independent experiments. *OK371>ACR1* 2 mM ATR, 44 μW/mm^2 n = 62 biologically independent animals over 3 independent experiments.

C. Locomotor activity of *OK371>KCR1-GS* flies before, during and after light actuation.

C1. *OK371/KCR1-GS* 0.5 mM ATR, 12 μW/mm^2 n = 47 biologically independent animals over 2 independent experiments. *OK371/KCR1-GS* 1 mM ATR, 12 μW/mm^2 n = 60 biologically independent animals over 3 independent experiments.

C2. *OK371/KCR1-GS* 0.5 mM ATR, 24 μW/mm^2 n = 46 biologically independent animals over 2 independent experiments. *OK371/KCR1-GS* 1 mM ATR, 24 μW/mm^2 n = 50 biologically independent animals over 2 independent experiments. *OK371/KCR1-GS* 2 mM ATR, 24 μW/mm^2 n = 44 biologically independent animals over 2 independent experiments.

C3. *OK371/KCR1-GS* 0.5 mM ATR, 44 μW/mm^2 n = 53 biologically independent animals over 3 independent experiments. *OK371/KCR1-GS* 1 mM ATR, 44 μW/mm^2 n = 44 biologically independent animals over 2 independent experiments. *OK371/-KCR1-GS* 2 mM ATR, 44 μW/mm^2 n = 41 biologically independent animals over 3 independent experiments.

D. Locomotor activity of *OK371>KCR1-ET* flies before, during and after light actuation.

D1. *OK371>KCR1-ET* 0.5 mM ATR, 12 μW/mm^2 n = 22 biologically independent animals over 1 independent experiment. *OK371>KCR1-ET* 1 mM ATR, 12 μW/mm^2 n = 48

biologically independent animals over 3 independent experiments. *OK371>KCR1-ET* 2 mM ATR, 12 µW/mm^2 n = 54 biologically independent animals over 3 independent experiments.

D2. *OK371>ACR1* 0.5 mM ATR, 24 µW/mm^2 n = 44 biologically independent animals over 2 independent experiments. *OK371>KCR1-ET* 1 mM ATR, 24 µW/mm^2 n = 58 biologically independent animals over 3 independent experiments. *OK371>KCR1-ET* 2 mM ATR, 24 µW/mm^2 n = 66 biologically independent animals over 3 independent experiments. *OK371>KCR1-ET* 0.5 mM ATR, 44 µW/mm^2 n = 63 biologically independent animals over 3 independent experiments.

D3. *OK371>KCR1-ET* 1 mM ATR, 44 µW/mm^2 n = 52 biologically independent animals over 3 independent experiments. *OK371>KCR1-ET* 2 mM ATR, 44 µW/mm^2 n = 66 biologically independent animals over 3 independent experiments. Additional statistical information for all panels is presented in Supplementary Dataset 1. Source data are provided as a Source Data file.

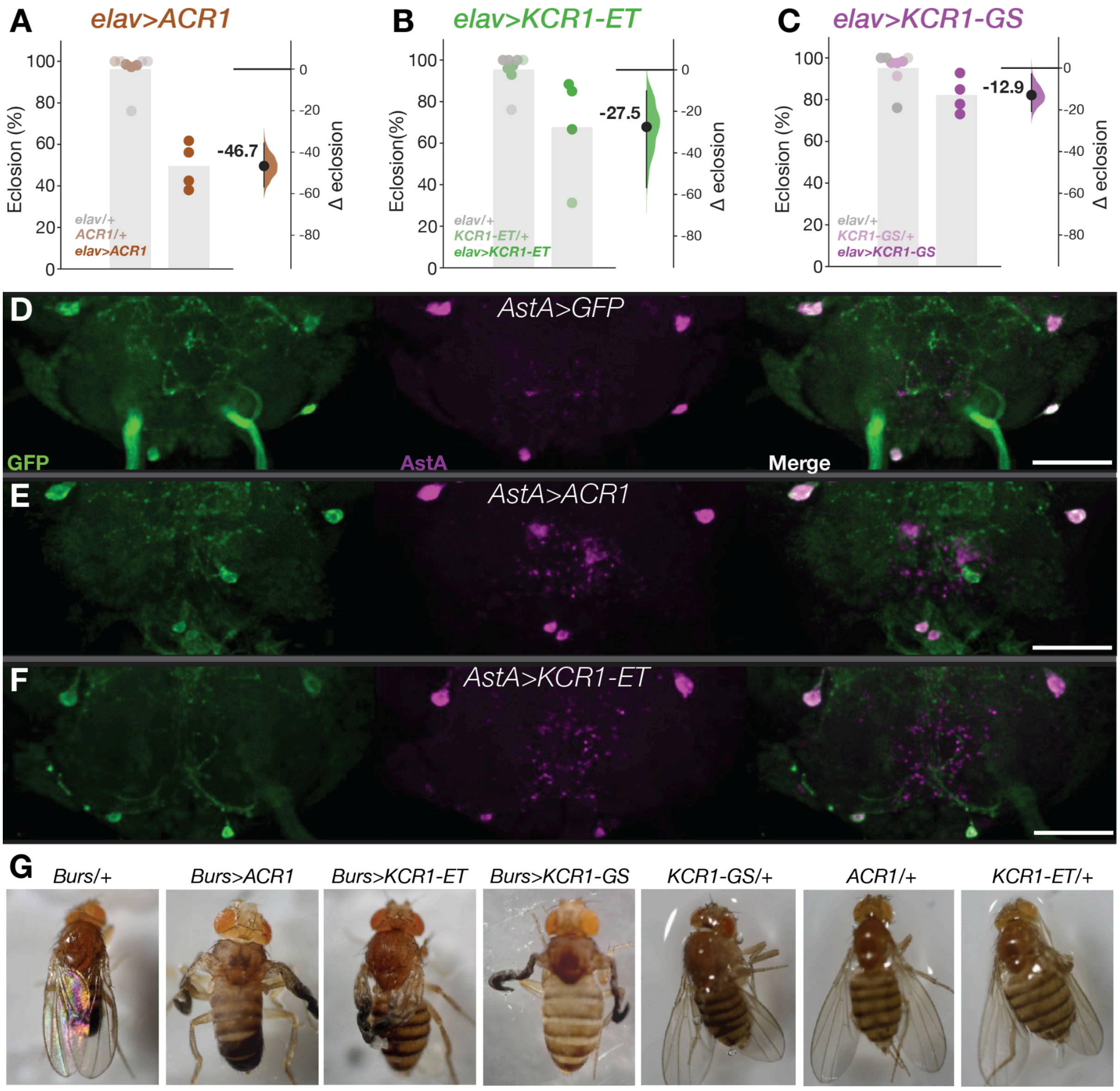

Figure S4: Toxicity is lower with KCR1 transgenes

A-C. Developmental lethality of dark-raised pan-neuronal *elav>ACR1* (**A**), *elav>KCR1-ET* (**B**) and *elav>KCR1-GS* (**C**) flies compared to genotypic controls. In each panel the left-hand axes display the percent of eclosed flies relative to the number of eggs laid. Each dot represents one experimental iteration. The right-hand axes show the Δ% between control and test conditions. The same *elav-Gal4/+* control flies were reused in each panel. Error bars represent the 95% CI. For ACR1 genotypic controls n = 514 biologically independent animals over 4 independent experiments. *Elav>ACR1* n = 299 biologically independent animals over 4 independent experiments. For KCR1-ET genotypic controls n = 462 biologically independent animals over 4 independent experiments. *Elav>KCR1-ET* n = 286 biologically independent animals over 4 independent experiments. For KCR1-GS genotypic controls n = 497 biologically independent animals over 4 independent experiments. *Elav>KCR1-GS* n = 259 biologically independent animals over 4 independent experiments.

D-F. Representative confocal images of fly brains expressing GFP or the respective opsin with *AstA-Gal4* in four cells in the subesophageal zone after six days of illumination. Anti-GFP is shown in green and anti-AstA in magenta. Scale bar = 50 um. For each genotype n = 1 biologically independent sample over 3 independent experiments.

G. Representative images of flies expressing the respective opsin with *Burs-Gal4* and control genotypes. Flies were exposed to green light (31 $\mu W/mm^2$) for all four days of metamorphosis. All of the *Burs>ACR1*, *Burs>KCR1-GS* and *Burs>KCR1-ET* flies displayed wing-expansion failure; the wings developed normally in all controls. For each genotype n = 100 biologically independent animals over 2 independent experiments. Additional statistical information is presented in Supplementary Dataset 1. Source data are provided as a Source Data file.

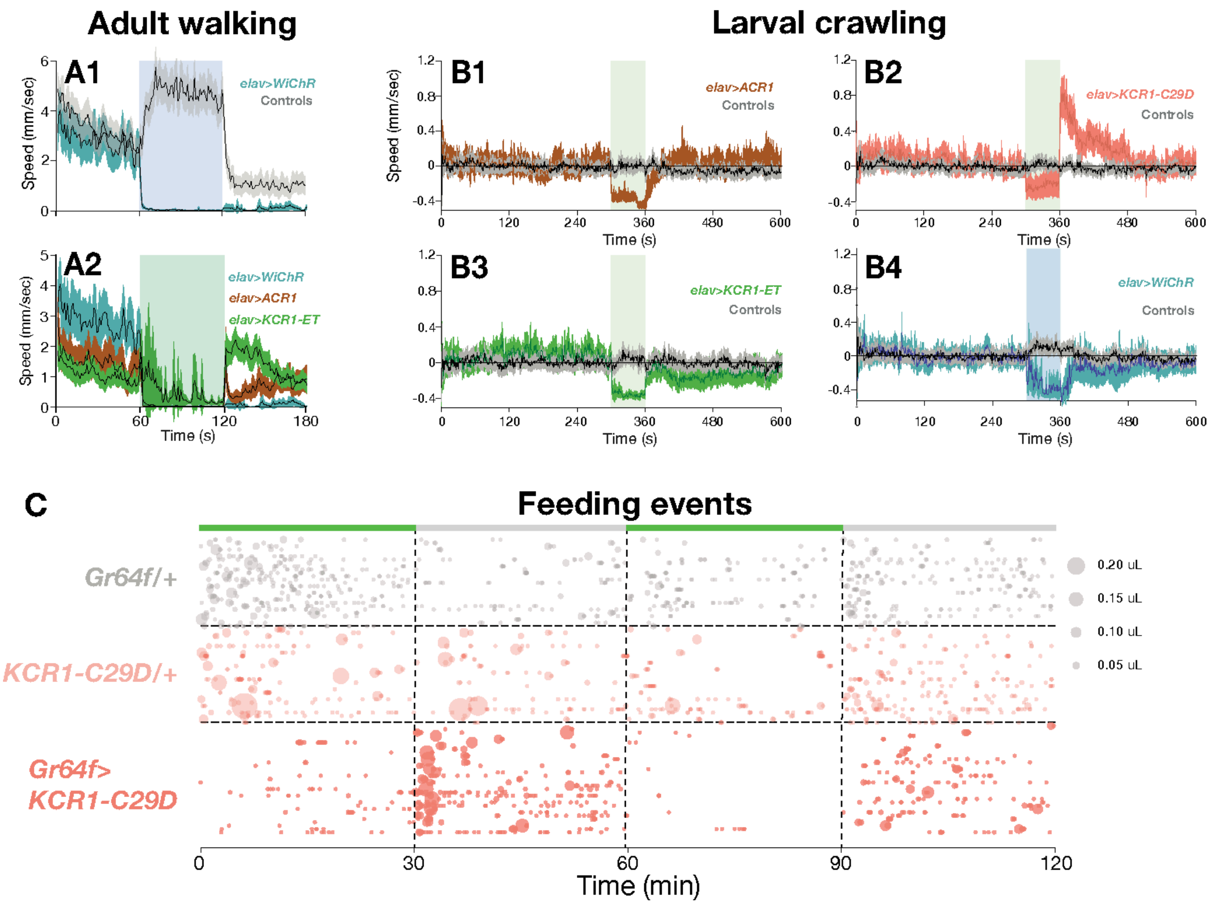

Figure S5: Inhibition of locomotion and feeding by KCR1-C29 and WiChR

A. Horizontal speed of *elav>WiChR* flies and genotypic controls before, during and after light actuation (top). Speed of the same *elav>WiChR* flies and those that expres *elav> ACR1* and *elav>KCR1-ET* (bottom). Error bars represent the 95% CI. Green illumination intensity was 24 μW/mm^2. Blue illumination was 24 μW/mm^2. For WiChR genotypic controls n = 126 biologically independent animals over 6 independent experiments. *Elav>WiChR* n = 55 biologically independent animals over 3 independent experiments. *Elav>KCR1-ET* n = 66 biologically independent animals over 4 independent experiments. *Elav>ACR1* n = 64 biologically independent animals over 4 independent experiments.

B. Locomotor assessment of *Drosophila* larvae before, during and after light illumination. Each respective opsin was expressed pan-neuronally with *elav-Gal4*. All crawling activity stopped upon light exposure in larvae expressing *ACR1* (**B1**), *KCR1* variants (**B2-3**) or *WiChR* (**B4**). In addition, larvae expressing *elav>KCR1-C29D* displayed curling behaviour and increased activity immediately after light exposure (**B2**). The *elav>WiChR* larvae displayed an approximately 60 s-long activity recovery delay following light exposure (**B4**). Respective genotypic controls are shown in grey. Error bands show 95% CI. Green light illumination intensity was 92 μW/mm^2. Blue light illumination was 27 μW/mm^2. ACR1 genotypic controls n = 58 biologically independent animals over 2 independent experiments. *Elav>ACR1* n = 39 biologically independent animals over 3 independent experiments. KCR1-C29D genotypic controls n = 73 biologically independent animals over 5 independent

experiments. *Elav>KCR1-C29D* n = 35 biologically independent animals over 2 independent experiments. KCR1-ET genotypic controls n = 60 biologically independent animals over 3 independent experiments. *Elav>KCR1-ET* n = 31 biologically independent animals over 2 independent experiments. WiChR genotypic controls n = 57 biologically independent animals over 2 independent experiments. *Elav>WiChR* n = 26 biologically independent animals over 3 independent experiments.

C. Feeding events of *Gr64f>KCR1-C29D* flies and genotypic controls in the presence and absence of light (24 μW/mm^2). Green bars indicate illumination epochs; the area of each bubble indicates volume of that feed event. KCR1-C29D genotypic controls n = 56 biologically independent animals over 3 independent experiments. *Gr64f>KCR1-C29D* n = 33 biologically independent animals over 3 independent experiments. Additional statistical information is presented in Supplementary Dataset 1. Source data are provided as a Source Data file.

Supplementary methods

C. elegans constructs

gBlocks (IDT) containing codon optimized cDNAs encoding either one of the opsins (ACR1, KCR1-ET, KCR1(GS) or KCR2-ET) that was tagged with YFP at its C-terminus (with three synthetic introns to enhance expression) was PCR amplified and ligated in the KpnI and EcoRI sites of *sdf-9P::mCherry* vector using the following primer sets:

ACR1-f and ACR-1-YFP-r for *sdf-9p::ACR1::YFP*; KCR-1-f and KCR-1-YFP-r for *sdf-9p::KCR1::YFP*; KCR-1-GS-f and KCR-1-GS-YFP-r for *sdf-9p::KCR1(GS)::YFP*; KCR-1-f and KCR-1-YFP-r for *sdf-9p::KCR2::YFP*.

Genomic DNA corresponding to pan-neuronal promoter (*snt-1p*) was PCR amplified from *C. elegans* genome using the following primers, snt-1P-FseI-F and snt-1P-AscI-R, and then ligated in the Fsel and Ascl sites, to generate *snt-1p::ACR1::YFP*, *snt-1p::KCR1::YFP, snt-1p::KCR1(GS)::YFP,* and *snt-1p::KCR2::YFP*. The plasmid was co-injected with *elt-2::mCherry* at 10ng/µl each into the gonads of adult N2 hermaphrodites by microinjector (InjectMan 4) to establish transgenic strains.

Live worm imaging by confocal fluorescence microscopy

For imaging experiments, L4 hermaphrodite worms were transferred to a glass slide and immobilized on 3% agarose pads using 2-3µl 1mg/µl levamisole diluted in M9 buffer. Images were then captured under a 100x objective. Multiple transgenic lines of each transgene were examined for fluorescent expression and localization patterns. Spinning disc confocal (SDC) microscopy was performed on a setup built around a Nikon Ti2 inverted microscope equipped with a Yokogawa CSU-W1 confocal spinning head, a Plan-Apo objective (100 × 1.45 NA), and a back-illuminated sCMOS camera (Prime 95B; Photometrics). Excitation light was provided by 488 nm/150 mW (Coherent) (for YFP) (power measured at optical fiber end) through DPSS laser combiner (iLAS system; Gataca systems). All image acquisition and processing were controlled by MetaMorph (Molecular Device) software. Images were acquired with exposure times in the 400–500 ms range.

Table S2: *C. elegans* genotypes and DNA constructs

Strain genotype	ID
C. elegans: yasEX257 [snt-1p::ACR1::YFP (10ng/ul); elt-2P::mCherry (10ng/ul)]	SAH743
C. elegans: yasEX259 [snt-1p::KCR1::YFP (10ng/ul); elt-2P::mCherry (10ng/ul)]	SAH745
C. elegans: yasEX260 [snt-1p::KCR2::YFP (10ng/ul); elt-2P::mCherry (10ng/ul)]	SAH746
C. elegans: yasEX261 [snt-1p::KCR1(GS)::YFP (10ng/ul); elt-2P::mCherry (10ng/ul)]	SAH747

Recombinant DNA constructs	ID
snt-1p::ACR1::YFP	RAI77
snt-1p::KCR1::YFP	RAI78
snt-1p::KCR1(GS)::YFP	RAI79
snt-1p::KCR2::YFP	RAI80
sdf-9p::mCherry	JB164

Recombinant DNA sequences	ID
acccttgGCTAGCgtcgacGGTACCggtagaaaaaATGAGTTCCATCACGTGC<br>GACCCTGCCATCTACGGCGAGTGGTCCCGAGAGAACCAGTTCTGCGTAGAGAA<br>GTCCTTGATAACCCTGGATGGAATTAAGTACGTCCAGCTGGTGATGGCAGTCG<br>TGTCAGCATGTCAAGTCTTTgtaagtttaaacagttcggtactaactaaccat<br>acatatttaaattttcagTTCATGGTGACAAGAGCACCGAAGGTTCCTTGGGA<br>AGCGATTTATTTGCCGACCACCGAAATGATTACCTATTCATTGGCCTTTACGG<br>GAAATGGTTACATTCGAGTCGCTAATGGCAAGTATCTTCCGTGGGCTCGAATG<br>GCATCTTGGCTTTGCACCTGCCCTATAATGCTTGGACTTGTATCCAACATGGC<br>CTTAGTAAAGTACAAATCAATCCCGCTTAACCCTATGATGATTGCCGCGTCTT<br>CTATATGTACCGTATTCGGAATAACGGCTTCCGTAGTATTGGATCCTCTTCAT<br>GTCTGGCTGTACTGCTTTATTTCGAGTATATTCTTTATATTTGAAATGGTCGT<br>AGCGTTTGCTATATTTGCTATCACGATCCACGATTTTCAGACGATAGGATCAC<br>CAATGTCGCTTAAAGTGGTTGAAAGACTCAAACTTATGCGAAgtaagtttaaa<br>catgattttactaactaactaatctgatttaaattttcagTTGTATTCTACGT<br>TTCCTGGATGGCCTATCCTATTCTCTGGTCATTCTCTTCCACGGGTGCTTGTA<br>TTATGTCGGAGAACACTTCTTCGGTTTTATATCTTTTGGGCGATGCTCTCTGT<br>AAAAACACGTATGGTATTCTGCTTTGGGCTACTACTTGGGGCCTCCTTAACGG<br>CAAATGGGACCGAGACTATGTTAAGGGCCGAAACGTGGATGGAGCCGCCGCCG<br>TGAGCAAGGGAGAGGAGCTGTTCACCGGAGTGGTGCCCATCCTGGTGGAGCTG<br>GATGGCGACGTGAACGGCCACAAGTTCTCGGTGAGCGGAGAGGGAGAGGGCGA<br>CGCCACCTACGGCAAGCTGACCCTGAAGTTCATCTGCACCACCGGCAAGCTGC<br>CCGTGCCGTGGCCAACCCTGGTGACCACCTTCGGCTACGGCCTGCAGTGCTTC<br>GCCCGCTACCCAGATCACATGAAGCAGCACGACTTCTTCAAGTCGGCCATGCC<br>GGAGGGATACGTGCAGGAGCGCACCATCTTCTTCAAGGATGACGGCAACTACA<br>AGACCCGCGCCGAGGTGAAGTTCGAGGGCGATACCCTGGTGAACCGCATCGAG<br>CTGAAGGGCATCGATTTCAAGGAGGACGGCAATATCCTGGGCCACAAGCTGGA<br>GTACAACTACAATAGCCACAACGTGTACATCATGGCCGACAAGCAGAAGAACG	RAI77_gBlock_ACR1

GCATCAAGGTTAATTTCAAGATCCGCCACAATATCGAGGATGGCTCCGTGCAG<br>CTGGCCGACCACTACCAGCAGAACACCCCGATTGGCGATGGACCCGTGCTGCT<br>gtaagtttaaacatatatatactaactaaccctgattatttaaattttcagGC<br>CAGACAATCACTACCTGAGCTACCAGTCCGCCCTGTCGAAGGACCCCAACGAG<br>AAGCGCGACCACATGGTGCTGCTGGAGTTTGTGACCGCCGCCGGAATTACCCT<br>GGGAATGGACGAGCTGTATAAGttctgctacgagaacgaggtgTAAGAATTCc<br>aactgagcgccggtcgcta	
acccttgGCTAGCgtcgacGGTACCggtagaaaaaATGCCATTCTACGACAGT<br>AGACCGCCGGAAGGTTGGCCAAAGGGTTCCATCAATGATATGGACTACCCGCT<br>CCTCGGTTCCATCTGCGCCGTCTGTTGCGTTTTCGTGGCGGGAAGTGGTATAT<br>GGATGCTGTACCGTTTAGATCTGGGTATGGGATACTCTTGTAAACCGTACAAA<br>TCGGGCCGAGCGCCAGAGGTCAATTCTCTGTCCGGAATTATATGTCTGCTTTG<br>CGGCACGATGTATGCGGCGAAATCATTTGATTTCTTTGACGGCGGAGGAACTC<br>CTTTCTCCCTGAACTGGTATTGGTATCTGGATTATGTGTTCACTTGTCCGCTG<br>CTGATCTTAGATTTTGCGTTCACATTGGACCTCCCACATAAGATTAGATACTT<br>CTTCGCTGTTTTTTTGACCCTCTGGTGCGGCGTTGCGGCGTTTGTTACACCGA<br>GTGCATACCGTTTCGCGTACTACGCATTGGGATGCTGCTGGTTCACCCCATTC<br>GCCCTCTCCCTTATGCGACACGTGAAAGAGCGATATTTAGTATACCCGCCGAA<br>GTGTCAGAGATGGCTCTTCTGGGCATGTGTGATATTCTTCGGTTTTTGGCCGA<br>TGTTTCCGATTTTATTCATATTCAGTTGGTTGGGTACTGGCCATATATCTCAA<br>CAGGCTTTCTACATAATCCACGCATTCTTGGACTTAACGTGTAAATCGATTTT<br>TGGCATATTGATGACTGTATTTCGTCTCGAGTTAGAGGAGCACACGGAAGTGC<br>AAGGACTGCCTCTTAATGAACCAGAAACCTTATCGGCTGCTGCGAAGTCTCGT<br>ATCACATCAGAGGGTGAATACATACCTCTGGATCAAATTGACATAAACGTTgc<br>cgccgccaagagcaggatcaccagcgagggcgagtacatcccccctggaccaga<br>tcgacatcaacgtgGTGAGCAAGGGAGAGGAGCTGTTCACCGGAGTGGTGCCC<br>ATCCTGGTGGAGCTGGATGGCGACGTGAACGGCCACAAGTTCTCGGTGAGCGG<br>AGAGGGAGAGGGCGACGCCACCTACGGCAAGCTgtaagtttaaacagttcggt<br>actaactaaccatacatatttaaattttcagGACCCTGAAGTTCATCTGCACC<br>ACCGGCAAGCTGCCCGTGCCGTGGCCAACCCTGGTGACCACCTTCGGCTACGG<br>CCTGCAGTGCTTCGCCCGCTACCCAGATCACATGAAGCAGCACGACTTCTTCA<br>AGTCGGCCATGCCGGAGGGATACGTGCAGGAGCGCACCATCTTCTTCAAGGAT<br>GACGGCAACTACAAGACCCGCGCCGAGGTGAAGTTCGAGGGCGATACCCTGGT<br>GAACCGCATCGAGCTGAAGGGCgtaagtttaaacatgattttactaactaact<br>aatctgatttaaattttcagATCGATTTCAAGGAGGACGGCAATATCCTGGGC<br>CACAAGCTGGAGTACAACTACAATAGCCACAACGTGTACATCATGGCCGACAA<br>GCAGAAGAACGGCATCAAGGTTAATTTCAAGATCCGCCACAATATCGAGGATG<br>GCTCCGTGCAGCTGGCCGACCACTACCAGCAGAACACCCCGATTGGCGATGGA<br>CCCGTGCTGCTGCCAGACAATCACTACCTGAGCTACCAGTCCGCCCTGTCGAA<br>GGACCCCAACGAGAAGCGCGACCACATGGTGCgtaagtttaaacatatatata<br>ctaactaaccctgattatttaaattttcagTGCTGGAGTTTGTGACCGCCGCC	RAI321_gBlock_<br>KCR1

GGAATTACCCTGGGAATGGACGAGCTGTATAAGttctgctacgagaacgaggt<br>gTGAGAATTCcaactgagcgccggtcgcta	
acccttgGCTAGCgtcgacGGTACCggtagaaaaaATGCCTTTTTACGATTCA<br>CGTCCTCCGGAAGGCTGGCCTAAGGGCTCCATTAACGATATGGACTATCCGCT<br>GTTGGGCTCAATTTGCGCTGTATGTTGCGTCTTCGTCGCTGGATCGGGTATCT<br>GGATGCTGTATCGACTTGATTTAGGTATGGGCTATTCCTGTAAGCCGTATAAA<br>TCAGGACGAGCACCGGAAGTGAACTCTCTTTCAGGCATAATATGTCTCCTTTG<br>TGGTACTATGTACGCCGCAAAGTCATTCGATTTTTTCGACGGAGGCGGCACTC<br>CGTTTTCGCTCAATgtaagtttaaacagttcggtactaactaaccatacatat<br>ttaaattttcagTGGTACTGGTATTTAGACTATGTATTCACTTGTCCACTGTT<br>AATCCTCGATTTCGCATTCACATTGGACCTTCCACACAAGATTCGATACTTTT<br>TTGCTGTTTTCTTAACACTCTGGTGTGGCGTGGCGGCCTTTGTAACTCCATCT<br>GCGTATCGATTCGCTTATTACGCACTCGGATGTTGCTGGTTCACACCTTTTGC<br>GCTCAGTCTGATGCGTCACGTTAAAGAGCGATATTTGGTGTATCCTCCTAAGT<br>GCCAACGATGGTTATTCTGGGCCTGTGTGATCTTTTTCGGCTTTTGGCCTATG<br>TTCCCGATACTCTTTATTTTTTCGTGGCTTGGAACCGGCCACATTTCGCAGCA<br>GGCATTTTACATTATTCACGCATTCCTCGACCTCACTTGTAAATCGATCTTTG<br>GAATACTTATGACTGTGTTTAGATTGGAACTGGAGGAGCATACCGAAGTACAG<br>GGCTTGCCATTGAATGAACCGGAGACGTTATCCACGGGAGGAGGTGGAGGATC<br>GGGTGGTGGAGGTTCAGGAGGCGGAGGCAGTGGCTCTACAGGAGGAGGAGGAG<br>GATCAGGAGgtaagtttaaacatgattttactaactaactaatctgatttaaa<br>ttttcagGAGGAGGATCAGGAGGAGGAGGATCAggaTCAGTGAGCAAGGGAGA<br>GGAGCTGTTCACCGGAGTGGTGCCCATCCTGGTGGAGCTGGATGGCGACGTGA<br>ACGGCCACAAGTTCTCGGTGAGCGGAGAGGGAGAGGGCGACGCCACCTACGGC<br>AAGCTGACCCTGAAGTTCATCTGCACCACCGGCAAGCTGCCCGTGCCGTGGCC<br>AACCCTGGTGACCACCTTCGGCTACGGCCTGCAGTGCTTCGCCCGCTACCCAG<br>ATCACATGAAGCAGCACGACTTCTTCAAGTCGGCCATGCCGGAGGGATACGTG<br>CAGGAGCGCACCATCTTCTTCAAGGATGACGGCAACTACAAGACCCGCGCCGA<br>GGTGAAGTTCGAGGGCGATACCCTGGTGAACCGCATCGAGCTGAAGGGCATCG<br>ATTTCAAGGAGGACGGCAATATCCTGGGCCACAAGCTGGAGTACAACTACAAT<br>AGCCACAACGTGTACATCATGGCCGACAAGCAGAAGAACGGCATCAAGGTTAA<br>TTTCAAGATCCGCCACAATATCGAGGATGGCTCCGTGCAGCTGGCCGACCACT<br>ACCAGCAGAACACCCCGATTGGCGATGGACCCGTGCTGCTgtaagtttaaaca<br>tatatatactaactaaccctgattatttaaattttcagGCCAGACAATCACTA<br>CCTGAGCTACCAGTCCGCCCTGTCGAAGGACCCCAACGAGAAGCGCGACCACA<br>TGGTGCTGCTGGAGTTTGTGACCGCCGCCGGAATTACCCTGGGAATGGACGAG<br>CTGTATAAGttctgctacgagaacgaggtgGAATTCcaactgagcgccggtcg<br>cta	RAI322_gBlock_<br>KCR1(GS)
acccttgGCTAGCgtcgacGGTACCggtagaaaaaATGCCATTCTACGACAGT<br>AGACCGCCGGAAGGTTGGCCACGAGGCTCCGTGAACGACATGGACTATCCGCT<br>TCTGGGCTCAATCTGTGCGATTTCTGCGATAGCTATTGCGGGATCGGGCATAT<br>GGATGTTATATCGATTAGACCTGGGAATGGGCTATTCTTGCAAACCGTATAAA	RAI323_gBlock_<br>KCR2

TCAGGTCGTGCTCCTGAAGTAAACTCTATTTCCGGAATAGTGTGCCTGCTCTG<br>CGGCACAATGTACGCAGCCAAATCGTTCGATTTTTTCGACGGTGGTGGAACTC<br>CATTTTCACTTAACTGGTACTGGTACTTAGATTATGTGTTCACCTGTCCTCTG<br>TTGATAGTTGATTTCGCGTTCACCCTGGACATTCCACAAAAGCTGAGATACTC<br>CATTGCCGTATTCGTCGCTCTGTGGTGTGCTGTAGCGGCGTTCGCTACACCAT<br>CGGCTTTTCGATTCGCGTATTATGCGCTCGGTTGCTGTTGGTTCATCCCTCTG<br>TCTCTGTTCCTTATACGAGACGTAAAAAAGCGTTACCAGGTTTATCCGCCAAA<br>GTGCCAGCGTCTGCTGTTTTGGGCCTGTGTTGTCTTCTTTGGATTCTGGCCTT<br>TGTTTCCATTGCTCTTTATCTTTTCGTGGCAGGGTTCTGGACACATCTCGCGT<br>CAAGCGTATTACATCATCCATGCTTTCCTTGACTTAGTATGCAAGTCTATATT<br>TGGATTTTTGATGACTTTCTTCCGATTAGAATTAGAGGAGCACACAGAAGTAC<br>AGGGTCTCCCACTGAAGGAACCGAAAGTCATGGACGCTGCGGCTAAATCCCGA<br>ATAACGTCTGAAGGAGAGTATATCCCATTGGATCAAATTGACATCAACGTAgc<br>cgccgccaagagcaggatcaccagcgagggcgagtacatcccctggaccaga<br>tcgacatcaacgtgGTGAGCAAGGGAGAGGAGCTGTTCACCGGAGTGGTGCCC<br>ATCCTGGTGGAGCTGGATGGCGACGTGAACGGCCACAAGTTCTCGGTGAGCGG<br>AGAGGGAGAGGGCGACGCCACCTACGGCAAGCTgtaagtttaaacagttcggt<br>actaactaaccatacatatttaaattttcagGACCCTGAAGTTCATCTGCACC<br>ACCGGCAAGCTGCCCGTGCCGTGGCCAACCCTGGTGACCACCTTCGGCTACGG<br>CCTGCAGTGCTTCGCCCGCTACCCAGATCACATGAAGCAGCACGACTTCTTCA<br>AGTCGGCCATGCCGGAGGGATACGTGCAGGAGCGCACCATCTTCTTCAAGGAT<br>GACGGCAACTACAAGACCCGCGCCGAGGTGAAGTTCGAGGGCGATACCCTGGT<br>GAACCGCATCGAGCTGAAGGGCgtaagtttaaacatgattttactaactaact<br>aatctgatttaaattttcagATCGATTTCAAGGAGGACGGCAATATCCTGGGC<br>CACAAGCTGGAGTACAACTACAATAGCCACAACGTGTACATCATGGCCGACAA<br>GCAGAAGAACGGCATCAAGGTTAATTTCAAGATCCGCCACAATATCGAGGATG<br>GCTCCGTGCAGCTGGCCGACCACTACCAGCAGAACACCCCGATTGGCGATGGA<br>CCCGTGCTGCTGCCAGACAATCACTACCTGAGCTACCAGTCCGCCCTGTCGAA<br>GGACCCCAACGAGAAGCGCGACCACATGGTGCgtaagtttaaacatatatata<br>ctaactaaccctgattatttaaattttcagTGCTGGAGTTTGTGACCGCCGCC<br>GGAATTACCCTGGGAATGGACGAGCTGTATAAGttctgctacgagaacgaggt<br>gTGAGAATTCcaactgagcgccggtcgcta	
ttttcaggaggacccttgGCTAGCgtcgacGGTACCggtagaaaaa	RAI215_ACR1-f
ctcagttgGAATTCTTAcacctcgt	RAI216_ACR-1-YFP-r
ttttcaggaggacccttgGCTAGCgtcgacGGTACCggtagaaaaaATGCCATTCTACGA	RAI217_KCR-1-f
ctcagttgGAATTCTCAcacctcgttctcgtagcagaaCTTATAC	RAI218_KCR-1-YFP-r

GCTAGCgtcgacGGTACCggtagaaaaaATGCCTTTTTACGATTC	RAI219_KCR-1-GS-f
ctcagttgGAATTCcacctcgttct	RAI220_KCR-1-GS-YFP-r
actgactgGGCCGGCCTTCCTTCAGAAGACGTGCTTTCCTTTTTC	RAI229_snt-1p-FseI-F
cctctagaGGCGCGCCGGTGACTGAAAGTTTGATTGATAAATGAA	RAI230_snt-1p-AscI-R
