# Supplementary material for: Kalium channelrhodopsins effectively inhibit neurons
Source: Nat Commun. 2024 Apr 24;15:3480. doi: 10.1038/s41467-024-47203-w (PMC11043423; doi:10.1038/s41467-024-47203-w)
Supplement: Supplementary file 3 — Description of Additional Supplementary Information [file 41467_2024_47203_MOESM3_ESM.pdf]

Description of Additional Supplementary Information

Kalium channelrhodopsins effectively inhibit neurons

Stanislav Ott[1], Sangyu Xu[2], Nicole Lee [1], Ivan Hong Hee Kean [1], Jonathan Anns[2,3], Danesha Devini Suresh [1], Zhiyi Zhang [1], Xianyuan Zhang [1], Raihanah Harion [4], Weiying Ye [5], Vaishnavi Chandramouli [4], Suresh Jesuthasan [4], Yasunori Saheki [4], Adam Claridge-Chang[1,2,4,6]

1. Program in Neuroscience and Behavioral Disorders, Duke-NUS Medical School, Singapore
2. Institute for Molecular and Cell Biology, A*STAR Agency for Science, Technology and Research, Singapore
3. School of Biological Sciences and Institute for Life Sciences, University of Southampton, UK
4. Lee Kong Chian School of Medicine, Nanyang Technological University, Singapore
5. Department of Pharmacy, National University of Singapore, Singapore
6. Correspondence. claridge-chang.adam@duke-nus.edu.sg

Supplementary Video 1. Each video shows *Drosophila* activity before, during and after opsin actuation in the Trumelan activity-monitoring assay (see Methods in the main manuscript). The video is centred on the tracked image of the fly, so the background is continuously updated. The initial 58 s of each video show flies being exposed to infrared illumination only, followed by 60 s of exposure to green light (λ 530 nm) illumination and a second epoch of 60 s infrared illumination. *OK371-Gal4* was used to drive opsin expression in motor neurons. In video SV1 the *OK371>ACR1* fly was maintained on food supplemented with 1 mM ATR (see Methods in the main manuscript) and a green light illumination intensity of 23.7 μW/mm^2 was used. Upon green light exposure (at the 58 s elapsed time point in each video) all opsin-bearing flies immediately fell on their back and remained in this position until the green light was switched off (1 min 58 s elapsed time point in each video). At the end of green light illumination (1 min 59 s elapsed time point in each video) flies regained their upright posture and displayed unimpaired locomotor activity. All videos were captured at 10 FPS.

Supplementary Video 2. The *OK371>KCR1-ET* fly was maintained on 1 mM ATR and illuminated with 23.7 μW/mm^2 of 530 nm light. Throughout the opsin actuation epoch, sporadic limb movement was observed in KCR1-expressing flies.

Supplementary Video 3. The *OK371>KCR1-GS* fly was maintained on 1 mM ATR and illuminated with 23.7 μW/mm^2 of 530 nm light.

Supplementary Video 4. An *OK371>ACR1* fly was maintained on 2 mM ATR food and illuminated with 44.6 μW/mm^2 of 530 nm light to actuate the opsin.

Supplementary Video 5. An *OK371>KCR1-ET* fly was maintained on 2 mM ATR food and illuminated with 44.6 μW/mm^2 of 530 nm light to actuate the opsin.

Supplementary Video 6. An *OK371>KCR1-GS* fly was maintained on 2 mM ATR food and illuminated with 44.6 μW/mm^2 of 530 nm light to actuate the opsin.

Supplementary Video 7. The video shows a *C. elegans* worm activity before, during and after opsin actuation in an open field arena. The coloured dots represent DeepLabCut key points. In the initial 10 s, the *snt1>ACR1* worm was exposed to infrared illumination only, followed by addition of 10 s of green light (λ 530 nm, 75 μW/mm^2) and a second 40 s epoch of infrared-only illumination. The *snt1-p* driver was used to express each opsin pan-neuronally. Prior to the experiment the worm was maintained on media containing 2mM ATR. Video was captured at 30 FPS. The worm stopped crawling and remained stationary during the opsin actuation period; shortly after light-off it resumed crawling behaviour.

Supplementary Video 8. Video tracking of a *snt1>KCR1-ET* worm before, during, and after illumination. (Conditions otherwise identical to Supplementary Video 7.)

Supplementary Video 9. Video tracking of a *snt1>KCR1-GS* worm before, during, and after illumination. (Conditions otherwise identical to Supplementary Video 7.)

Supplementary Dataset 1

The Supplementary Dataset 1 (an **.xlsx** spreadsheet) lists the genotypic and statistical information for all experiments described within the manuscript. The Figure and Panel columns index the respective information to the figures in the manuscript. The Genotype 1 and Genotype 2 columns provide the genotype for the respective "Test" and "Control" genotypes. The Assay and Protocol columns contain information on the assay used and the type of experiment performed. The sample size (N) columns display the total number samples used in each experiment and the experimental iterations (N iterations) columns display the number of repetitions for the respective experiment. The estimation statistics columns show the mean difference effect size delta (Δ Effect size) between the control and test genotypes with 95% confidence intervals (CI) with the corresponding P value (P).
