# Supplementary material for: Kalium channelrhodopsins effectively inhibit neurons
Source: Nat Commun. 2024 Apr 24;15:3480. doi: 10.1038/s41467-024-47203-w (PMC11043423; doi:10.1038/s41467-024-47203-w)
Supplement: Supplementary file 14 — Reporting Summary [file 41467_2024_47203_MOESM14_ESM.pdf]

nature portfolio

Corresponding author(s): Adam Claridge-Chang

Last updated by author(s): Feb 13 2024

Reporting Summary

Nature Portfolio wishes to improve the reproducibility of the work that we publish. This form provides structure for consistency and transparency in reporting. For further information on Nature Portfolio policies, see our Editorial Policies and the Editorial Policy Checklist.

Statistics

For all statistical analyses, confirm that the following items are present in the figure legend, table legend, main text, or Methods section.

n/a	Confirmed	
☐	☒	The exact sample size (*n*) for each experimental group/condition, given as a discrete number and unit of measurement
☐	☒	A statement on whether measurements were taken from distinct samples or whether the same sample was measured repeatedly
	☒	The statistical test(s) used AND whether they are one- or two-sided<br>*Only common tests should be described solely by name; describe more complex techniques in the Methods section.*
☒	☐	A description of all covariates tested
☐	☒	A description of any assumptions or corrections, such as tests of normality and adjustment for multiple comparisons
☐	☒	A full description of the statistical parameters including central tendency (e.g. means) or other basic estimates (e.g. regression coefficient) AND variation (e.g. standard deviation) or associated estimates of uncertainty (e.g. confidence intervals)
	☒	For null hypothesis testing, the test statistic (e.g. *F*, *t*, *r*) with confidence intervals, effect sizes, degrees of freedom and *P* value noted<br>*Give P values as exact values whenever suitable.*
☒	☐	For Bayesian analysis, information on the choice of priors and Markov chain Monte Carlo settings
☒	☐	For hierarchical and complex designs, identification of the appropriate level for tests and full reporting of outcomes
☐	☒	Estimates of effect sizes (e.g. Cohen's *d*, Pearson's *r*), indicating how they were calculated

Our web collection on statistics for biologists contains articles on many of the points above.

Software and code

Policy information about availability of computer code

Data collection	C. elegans behavior was recorded by using DeepLabCut (DLC) pose-estimation neural-network software (DOI: 10.1038/s41593-018-0209-y). D. melanogaster larvae electrophysiology recordings were performed by using pCLAMP 10 software (Molecular Devices). D. melanogaster behavior was recorded and processed in real time by using CRITTA tracking software (DOI: 10.1016/j.cub.2014.03.073).
Data analysis	C. elegans behavior was analyzed using a custom script written in Python 3.10. D. rerio behavior data collection and analysis was carried out using Fiji (DOI: 10.1038/nmeth.2019) and custom scripts written in Python 3.10 as previously described (DOI 10.1186/s12915-017-0430-2). D. melanogaster behavioral data were analyzed using custom analysis software written in Python 3.10. Confocal image analysis was performed using Fiji (DOI: 10.1038/nmeth.2019). Data and code are deposited at: https://doi.org/10.5281/zenodo.10648742

For manuscripts utilizing custom algorithms or software that are central to the research but not yet described in published literature, software must be made available to editors and reviewers. We strongly encourage code deposition in a community repository (e.g. GitHub). See the Nature Portfolio guidelines for submitting code & software for further information.

Data

Policy information about availability of data

All manuscripts must include a data availability statement. This statement should provide the following information, where applicable:

- Accession codes, unique identifiers, or web links for publicly available datasets
- A description of any restrictions on data availability
- For clinical datasets or third party data, please ensure that the statement adheres to our policy

All data and code that support the findings of this study are available from the Zenodo data repository at: https://doi.org/10.5281/zenodo.10648742

Research involving human participants, their data, or biological material

Policy information about studies with human participants or human data. See also policy information about sex, gender (identity/presentation), and sexual orientation and race, ethnicity and racism.

Reporting on sex and gender	NA
Reporting on race, ethnicity, or other socially relevant groupings	NA
Population characteristics	NA
Recruitment	NA
Ethics oversight	NA

Note that full information on the approval of the study protocol must also be provided in the manuscript.

Field-specific reporting

Please select the one below that is the best fit for your research. If you are not sure, read the appropriate sections before making your selection.

☒ Life sciences ☐ Behavioural & social sciences ☐ Ecological, evolutionary & environmental sciences

For a reference copy of the document with all sections, see nature.com/documents/nr-reporting-summary-flat.pdf

Life sciences study design

All studies must disclose on these points even when the disclosure is negative.

Sample size	Sample sizes were determined based on established practices in the field {Cumming G (2013). Understanding the new statistics. Routledge, UK). Sample sizes for each dataset and other statistical information are presented in the respective figure legends and the supplementary table 1.
Data exclusions	No data were excluded from the analyses
Replication	Data were replicated at least once on a separate occasion, for behavioral data combined results were plotted in the manuscript. All attempts at replication were successful.
Randomization	For all experiments the genotypes tested were randomized on a daily basis.
Blinding	Blinding was not relevant for behavioral experiments. The experiment was recorded and evaluated automatically by the CRITTA software. Experimental manipulation due to implicit bias was not possible without actively tampering with the raw data. Blinding was not done for immunostaining experiments becasue we performed fluorimetry quantification.

Reporting for specific materials, systems and methods

We require information from authors about some types of materials, experimental systems and methods used in many studies. Here, indicate whether each material, system or method listed is relevant to your study. If you are not sure if a list item applies to your research, read the appropriate section before selecting a response.

Materials & experimental systems

n/a	Involved in the study	
☐	☒	Antibodies
☐	☒	Eukaryotic cell lines
☒	☐	Palaeontology and archaeology
☐	☒	Animals and other organisms
☒	☐	Clinical data
☒	☐	Dual use research of concern
☒	☐	Plants

Methods

n/a	Involved in the study	
☒	☐	ChIP-seq
☒	☐	Flow cytometry
☒	☐	MRI-based neuroimaging

Antibodies

Antibodies used

N2A staining;
Chicken anti GFP (Abcam ab13970, RRID AB_300798, 1;2000 v/v dilution)
D melanogaster staining;
Cockroach anti allatostatin (Ast7) (DSHB SFl0, 1;2 v/v dilution), Mouse anti discs-large 3 (DSHB 4F3)
Mouse anti BRP (DSHB nc8, RRID AB_23148662, 1;50 v/v dilution), Mouse anti-Dlg (4F3, DSHB, RRIDAB_528203) at 1;50 v/v dilution)
Chicken anti GFP (Abcam ab13970, RRID AB_300798, 1;2000 v/v dilution),
Alexa 488 goat anti-chicken (A-11039 Thermo Fisher Scientific, RRID AB_2534096, 1;1000 v/v dilution)
Alexa 647 donkey anti-mouse (715-605-151, Jackson ImmunoResearch, RRID AB_2340863, 1;500 v/v dilution).
Alexa 568 goat anti-mouse (A-11004 Thermo Fisher Scientific, RRID; AB_2534072, 1;500 v/v dilution)

Validation

All validation data and statements can be found on the manufacturer's website. Citations to the relevant publications have been provided in the manuscript

Eukaryotic cell lines

Policy information about cell lines and Sex and Gender in Research

Cell line source(s)

S2 cells were obtained from the Drosophila Genome Research Center. The N2a cells were a gift from the Jun Nishiyama lab who obtained the cells from the American Type Culture Collection (ATCC).

Authentication

Authentication of the original batch was performed by ATCC. No authentication was performed afterwards

Mycoplasma contamination

N2a cells were free of mycoplasma contamination. No check was performed on S2 cells

Commonly misidentified lines
(See ICLAC register)

No commonly misidentified lines were used in this study

Animals and other research organisms

Policy information about studies involving animals; ARRIVE guidelines recommended for reporting animal research, and Sex and Gender in Research

Laboratory animals

Drosophila melanogaster 3rd instar larvae and 5-10d old adults. Danio rerio F1embryos. Caenorhabditis elegans L4

Wild animals

No wild animals were used in the study

Reporting on sex

Both Drosophila males and females were used for behavioral experiments at an equal ratio. Drosophila brain dissections were performed with female flies. ~~NA for~~ C elegans and D rerio ~~experiments~~

Field-collected samples

No field collected samples were used in the study

Ethics oversight

D rerio experiments were performed in accordance with guidelines approved by the Institutional Animal Care and Use Committee of A-STAR Biopolis, Singapore. No ethical approvals were required for D. melanogaster and C. elegans studies.

Note that full information on the approval of the study protocol must also be provided in the manuscript.
